# Do we know enough about the effect of low-dose computed tomography screening for lung cancer on survival to act? A systematic review, meta-analysis and network meta-analysis of randomised controlled trials

**DOI:** 10.1186/s41512-019-0067-4

**Published:** 2019-11-28

**Authors:** Huiqin Yang, Jo Varley-Campbell, Helen Coelho, Linda Long, Sophie Robinson, Tristan Snowsill, Ed Griffin, Jaime Peters, Chris Hyde

**Affiliations:** 1 Peninsula Technology Assessment Group (PenTAG), University of Exeter Medical School, Exeter, UK; 2 Exeter Test Group, University of Exeter Medical School, Exeter, UK

**Keywords:** Lung neoplasms, Mass screening, Early detection of cancer, Tomography, X-ray computed, Tomography, Spiral computed, Review, Systematic

## Abstract

**Background:**

Diagnosis of lung cancer frequently occurs in its later stages. Low-dose computed tomography (LDCT) could detect lung cancer early.

**Methods:**

Our objective was to estimate the effect of LDCT lung cancer screening on mortality in high-risk populations. A systematic review of randomised controlled trials (RCTs) comparing LDCT screening programmes with usual care (no screening) or other imaging screening programme (such as chest X-ray (CXR)) was conducted. RCTs of CXR screening were additionally included in the network meta-analysis. Bibliographic sources including MEDLINE, Embase, Web of Science and the Cochrane Library were searched to January 2017. All key review steps were done by two persons. Quality assessment used the Cochrane Risk of Bias tool. Meta-analyses were performed.

**Results:**

Four RCTs were included. More will provide data in the future. Meta-analysis demonstrated that LDCT screening with up to 9.80 years of follow-up was associated with a statistically non-significant decrease in lung cancer mortality (pooled relative risk (RR) 0.94, 95% confidence interval (CI) 0.74 to 1.19; *p* = 0.62). There was a statistically non-significant increase in all-cause mortality. Given the considerable heterogeneity for both outcomes, the results should be treated with caution.

Network meta-analysis including the four original RCTs plus two further RCTs assessed the relative effectiveness of LDCT, CXR and usual care. The results showed that in terms of lung cancer mortality reduction LDCT was ranked as the best screening strategy, CXR screening as the worst strategy and usual care intermediate.

**Conclusions:**

LDCT screening may be effective in reducing lung cancer mortality but there is considerable uncertainty: the largest of the RCTs compared LDCT with CXR screening rather than no screening; there is imprecision of the estimates; and there is important heterogeneity between the included study results. The uncertainty about the effect on all-cause mortality is even greater. Maturing trials may resolve the uncertainty.

## Introduction

Lung cancer is the most common cancer in the world with 1.8 million new cases diagnosed in 2012 [1]. Lung cancer was attributable to 5.4% of the total number of deaths in the European Union (2013), equating to more than a quarter of a million people (268,744 people) or 55.2 deaths per 100,000 inhabitants [2]. Overall, the prognosis for long-term survival with lung cancer is poor. Net survival for adults (aged 15 to 99) in England and Wales in 2010–2011 was 32.1% at 1 year, 9.5% at 5 years and 4.9% at 10 years [3]. The main cause of lung cancer is smoking, so primary prevention remains the priority [4].

However, prognosis is related to stage at diagnosis. The 1-year survival rate is over 80% when lung cancer is diagnosed in stage I, but under 20% when diagnosed in stage IV [5], this being due to the ability to treat surgically with curative intent at early stages. Also few lung cancers present in their early stages, 25% in the UK National Lung Cancer Audit annual report 2016 [6]. Together, these facts suggest that there may be an opportunity to use secondary prevention by screening to increase the number of cancers identified at an early stage.

Over several decades, a number of potential screening tests for lung cancer have been investigated including chest x-ray (CXR) and sputum cytology. Neither of these has been found to be effective [7]. As computed tomography (CT) has developed, offering improved images at lower radiation dosage, so low-dose CT (LDCT) has become the test offering the greatest potential for effective and cost-effective screening for lung cancer with much research devoted to investigating this [7].

The National Lung Screening Trial (NLST) has been particularly influential comparing LDCT screening with CXR [8]. 53,454 persons at high risk for lung cancer in 33 US medical centres were randomised from August 2002 to April 2004, 26,722 to three annual screenings of LDCT and 26,732 to single-view posteroanterior CXR. All participants were followed to 31/12/2009. Investigators concluded that “screening with LDCT reduces mortality from lung cancer”. The US Preventive Services Task Force agreed with this and in 2013 changed their negative recommendation for screening for lung cancer screening to a positive one [9].

In the UK, population lung cancer screening is currently not carried out by the NHS, based on the recommendation of the UK National Screening Committee in July 2006 when they last assessed whether lung cancer screening should be recommended for adult cigarette smokers. They concluded that it should not be recommended but should be reviewed in 2015/2016. The research we report was commissioned to update the systematic review which underpinned the last guidance [10, 11].

## Methods

Our objective was to evaluate the clinical effectiveness of screening programmes for lung cancers with LDCT in high-risk populations using a systematic review, meta-analysis and network meta-analysis of RCTs. The wider project also considered cost-effectiveness. The systematic review was registered (PROSPERO CRD42016048530). All aspects of the work were undertaken in accordance with a pre-specified protocol [12] with some minor recorded exceptions. These involved an expansion of the range of outcomes we abstracted data on, searching some different websites to those originally specified and being more precise about what constituted poor study quality in the investigation of heterogeneity. The work was commissioned by the NIHR on behalf of the UK NHS to inform a future decision by the UK National Screening Committee on LDCT screening for lung cancer. The complete health technology assessment has recently been published [13].

We searched MEDLINE, MEDLINE In-Process, Embase, PsycINFO (all via Ovid), Web of Science (Thomson Reuters), CDSR and CENTRAL (via The Cochrane Library) and CINAHL (EBSCO) from 2004 to January 2017. (Additional file 1: Table S1. MEDLINE search strategy). Literature prior to 2004 was identified via the 2006 health technology assessment by the Aberdeen Health Technology Assessment Group [10] which this research project was commissioned to up-date. Other published and unpublished literature was identified from systematic searches of electronic sources, citation chasing, consultation with experts in the field and reference checking of relevant systematic reviews.

In the main systematic review and meta-analysis, we included LDCT lung cancer screening programme RCTs involving populations at high risk of lung cancer. Any definition of high risk was eligible. LDCT screening programmes included both single and multiple rounds. The eligible comparators were no screening or other imaging technology screening programmes (such as CXR). RCTs evaluating the effectiveness of CXR but not LDCT were also included in the network meta-analysis. The outcomes of interest for this analysis were lung cancer mortality and all-cause mortality.

Two researchers independently screened the titles and abstracts of all reports identified by the search strategy. Full-text papers were subsequently obtained and screened in the same way. Data extraction and quality assessment were undertaken by one researcher and checked by a second. The risk of bias of included studies was assessed using the Cochrane Risk of Bias tool [14]. We also considered underpowered sample size for important outcomes and significant baseline differences between study arms on important characteristics.

All data were tabulated and primarily considered in a narrative review. DerSimonian and Laird random-effects models meta-analyses were performed to pool the estimates of effect [15]. We restricted the meta-analysis to RCTs with at least 5 years follow-up consistent with the primary outcome in NLST. A random-effects approach was pre-specified as part of the protocol development process; a fixed approach was not favoured as it was thought highly unlikely that chance alone would account for differences between the results of included studies. Statistical heterogeneity was assessed using the I^2^ statistic. Based on the advice in the Cochrane handbook, 30% to 50% was categorised as moderate heterogeneity; 50% upwards as substantial heterogeneity [16]. We considered the following factors for the exploration of heterogeneity: quality of trials (particularly adequacy of randomisation), nature of interventions (e.g. frequency of LDCT screening), and nature of control groups (e.g. best available care such as CXR screening or usual care).

Network meta-analysis was performed to assess the relative effectiveness of three screening strategies (LDCT, usual care and CXR). A multivariate random-effects meta-analysis using restricted maximum likelihood approach was performed [17]. We estimated the relative ranking probability of each intervention and obtained the treatment hierarchy of competing interventions using rankogram, surface under the cumulative ranking curve and mean ranks [18]. The probability was estimated using a Bayesian model with flat priors, under the assumption that the posterior distribution of the parameter estimates was approximated by a normal distribution with mean and variance equal to the frequentist estimates and variance–covariance matrix [17]. In order to assess the presence of inconsistency, both consistency and inconsistency models were fit for data. We used the design-by-treatment model to check the assumption of consistency in the entire network [19]. This provides a robust approach to assess the consistency of the network being constructed.

Statistical analyses were performed using the ‘metan’ ‘mvmeta’ and ‘network’ commands in Stata 14 (StataCorp LLC, Texas) [20, 21].

## Results

**Fig. 1:**
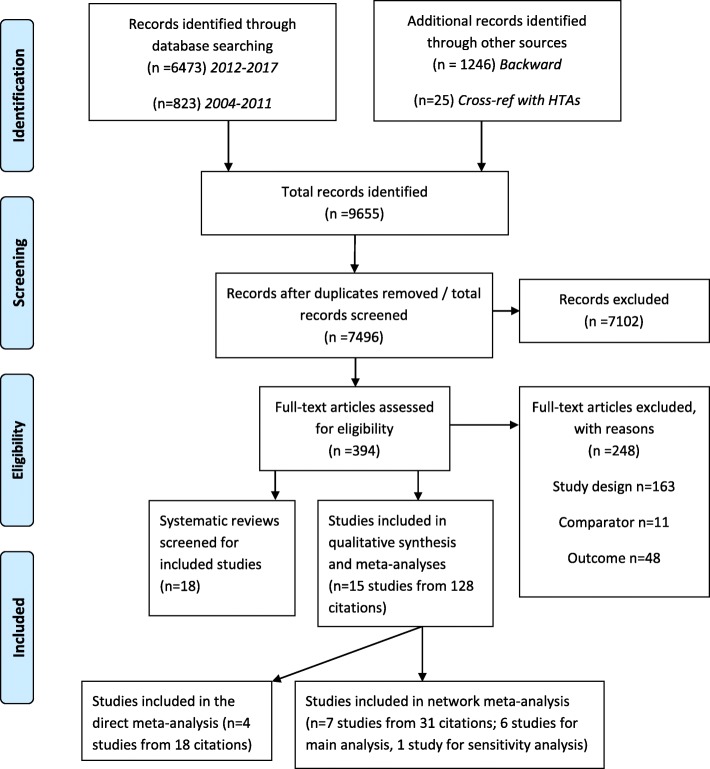
PRISMA diagram

From 9655 records identified in the searches, four RCTs were included in the meta-analysis of mortality data [8, 22–25] and a further two RCTs in the network meta-analysis [26–28] (Fig. 1). One further RCT was used in a sensitivity analysis of the network meta-analysis [29].

The characteristics of the included studies are shown in Table 1. Concerning the LDCT trials, most were conducted in Europe and the USA. There was variation between the LDCT programmes, but typically they involved 3–5 rounds of screening over 3 to 6.5 years. The nature of high-risk participants also varied but was usually defined in terms of age and current and past smoking history. Of the trials NLST stands apart, not just in terms of size with over 50,000 participants, but by LDCT being compared to CXR screening rather than no screening [8].

**Table 1:** Characteristics of included studies

Study	Country	Recruitment time	Screening programme	Comparator	Sample size, N	Participants age (yrs)	Number of screening rounds	Screening times and interval (yrs)	Duration of follow-up (mean/ median)
Direct meta-analysis and network meta-analysis – RCTs of LDCT screening									
DANTE [22]	Italy	03/2001 to 02/2006	LDCT, medical exam and one CXR	No screening, medical exam and one CXR	2,811 (2,400 planned)	60-74	5 vs 0	T0, T1, T2, T3, T4	At 12/2012 median 6 yrs 3.5 months
DLCST [23]	Denmark	10/2004 to 03/2006	LDCT	No screening	4,104	50-70	5 vs 0	T0, T1, T2, T3, T4	Median: 9.47 yrs vs 9.53 yrs (planned 10yrs)
MILD [24]	Italy	09/2005 to 09/2011	LDCT (annual and biannual), smoking cessation, pulmonary function test, blood sample	No screening, smoking cessation, pulmonary function test, blood sample	4,099 (10,000 planned)	>49	10 or 5 vs 0	T0, T1, T2, T3, T4, T5, T6, T7, T8, T9 vs T0, T2, T4, T6, T8	Median 7.3 yrs
NLST [8, 25]	USA	08/2002 to 04/2004	LDCT	CXR	53,454	55-74	3 vs 3	T0, T1, T2	Median 6.5 yrs
Network meta-analysis (main) – RCTs of CXR screening									
Czech [26, 27]	Czech republic	06/1976 to 06/1977	Intensive CXR, sputum cytology	Single CXR, sputum cytology	6,346	40-64	6 vs 1	T0, T0.5, T1, T1.5, T2, T2.5, T3 vs T0, T3. Further follow-up CXRs (no sputum) T4, T5,T6 in both arms	15 yrs all participants
MAYO [28]	USA	08/1971 to NR (screening ended 07/1976)	Intensive CXR, sputum cytology	Usual care (recommended an annual CXR and sputum cytology)	9,211	>45	18 vs unknown	T0.3, T0,7, T1, T1.3, T1.7, T2.0, T 2.3, T2.7, T3, T3.3, T3.7, T4, T4.3, T4.7, T5, T5.3, T5.7, T 6 (4 monthly) vs unknown	Median 20.5 years
Network meta-analysis (sensitivity) – RCTs of CXR screening (post hoc defined high risk sub-group of larger RCT)									
PLCO [29]	USA	1993 to 2001	CXR	No screening	154,901 (30,321 NLST eligible subgroup)	55-74	4 vs 0	T0, T1, T2, T3	6 yrs all participants NLST eligible subgroup

Abbreviations: *CXR* chest X-ray, *LDCT* low-dose computed tomography, *RCT* randomised controlled trial, *N* not reported, *yrs* years

Looking at the 12 included RCTs in the qualitative review (Additional file 1: Table S2) reveals that although only four RCTs currently contribute mortality data to the direct meta-analysis, there are others [ITALUNG [30], German Lung Cancer Screening Intervention trial (LUSI) [31], NEderlands Leuvens Longkanker Screenings ONderzoek (NELSON) [32, 33]] which started between 2001 to 2010 and will be maturing in the near future. In addition, the UK Lung Cancer Screening trial (UKLS) [34] has indicated that it will incorporate its mortality data with the NELSON study.

The two additional trials [26–28] for the network meta-analysis compared intensive screening with CXR and sputum cytology over 3 to 6 years with usual care involving occasional CXR examination. The frequency of screening in the intervention arms was much more frequent than the LDCT RCTs, with CXR examinations two or three times a year. The RCTs were done in the Czech Republic [26, 27] and the USA [28] in the 1970s and consequently benefit from long follow up. A third RCT of CXR screening conducted in the USA in the 1990s, Prostate, Lung, Colorectal and Ovarian cancer screening trial (PLCO) [29], could not be included because the majority of subjects were low risk. We did however include a post-hoc high-risk sub-group analysis of this trial in a sensitivity analysis as this subgroup (NLST-eligible subgroup involving high-risk participants) was relevant to our research question. It compared four annual rounds of CXR screening with no screening.

The majority of the LDCT included trials were judged to be of moderate to high quality, although allocation concealment was consistently poorly addressed. One RCT, Multicentric Italian Lung Detection project (MILD) [24], was however judged to be of much poorer quality with a particularly marked risk of bias arising from lack of clarity about randomisation, accompanied by marked imbalances in some of the baseline characteristics (Additional file 1: Table S3).

The additional RCTs for the network meta-analysis were of slightly poorer methodological quality than most of the LDCT RCTs, with greater lack of clarity about loss to follow-up and absence of power calculations. A mitigating factor may be that standards for reporting RCTs were not well established in the 1970s when the studies were conducted with the first Consolidated Statement of Reporting of Trials version being published in 1996 [35]. The PLCO main trial [29] was of similar quality to the LDCT RCTs, but the NLST sub-group study admitted very limited power to detect small differences in mortality and was only able to demonstrate baseline equivalence for a small number of characteristics (Table 2).

**Table 2:** Quality assessment of included studies

Study	Random sequence generation (selection bias)	Allocation concealment (selection bias)	Blinding of participants and personnel (performance bias)	Blinding outcome assessment (detection bias)	Incomplete outcome data (attrition bias)	Selective reporting (reporting bias)	Other risk of bias (power and baseline imbalance)
Direct meta-analysis and network meta-analysis—RCTs of LDCT screening							
DANTE [22]	Low	Unclear	Low	Low	Low	Low	Low and low
DLCST [23]	Low	Unclear	Low	Low	Low	Low	Low and low
MILD [24]	Unclear	Unclear	Low	Low	Low	Low	Inadequate and inadequate
NLST [8, 25]	Low	Unclear	Low	Low	Low	Low	Low and low
Network meta-analysis (main)—RCTs of CXR screening							
Czech [26, 27]	Low	Unclear	Low	Unclear	Unclear	Low	Unclear and unclear
MAYO [28, 36]	Low	Unclear	Low	Low	Unclear	Low	Unclear and low
Network meta-analysis (sensitivity)—RCTs of CXR screening (post hoc defined high risk sub-group of larger RCT)							
PLCO[29]	Low	Unclear	Low	Low	Low	Low	Inadequate and unclear

*CXR* chest x-ray, *LDCT* low-dose computed tomography, *RCT* randomised controlled trial, *N* not reported, *yrs* years

**Fig. 2:**
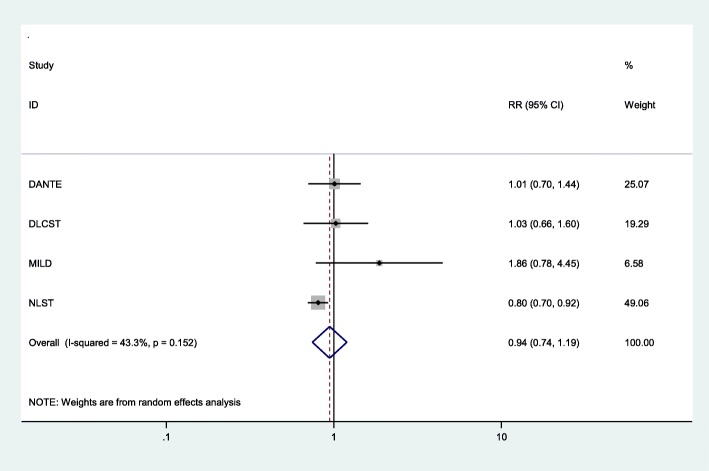
Lung cancer mortality—overall results

The direct meta-analysis showed that LDCT screening was associated with a statistically non-significant decrease in lung cancer mortality (pooled RR 0.94, 95% CI 0.74 to 1.19; *p* = 0.62) with up to 9.80 years of follow-up when compared with controls (Fig. 2). A moderate level of heterogeneity was observed in the magnitude of effects (*I*^2^ = 43.3%), given which the results should be treated with caution. A range of potential sources for heterogeneity were investigated. When removing the poor quality trial (MILD) [24], sensitivity analysis demonstrated a statistically significant decrease in lung cancer mortality (pooled RR 0.85, 95% CI 0.74 to 0.98; *p* = 0.02) in favour of LDCT screening compared with controls (Additional file 1: Figure S1). A considerable reduction in heterogeneity was observed (*I*^2^ = 6.9%).

**Fig. 3:**
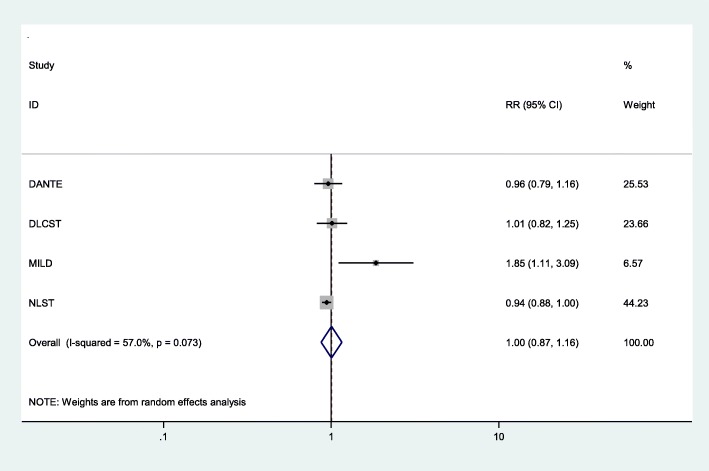
All-cause mortality—overall results

The direct meta-analysis also showed that, compared with controls, LDCT screening demonstrated a statistically non-significant increase in all-cause mortality outcome (pooled RR 1.01, 95% CI 0.87 to 1.16; *p* = 0.95) with up to 9.80 years of follow-up (Fig. 3). Likewise, given the substantial heterogeneity (*I*^2^ = 57.0%) detected between studies, the results from this pooled analysis should be treated with caution. We also investigated the potential sources of heterogeneity. When removing the low-quality trial (MILD) [24], sensitivity analysis showed that LDCT screening demonstrated a borderline statistically non-significant decrease in all-cause mortality (pooled RR 0.95, 95% CI 0.89 to 1.00; *p* = 0.05) compared with controls (Additional file 1: Figure S2). The level of heterogeneity was also considerably reduced (*I*^2^ = 0%), suggesting that variation in trial quality could be a potential source of heterogeneity between studies.

Network meta-analysis assessed the relative effectiveness of LDCT, usual care and CXR screening. The main network consisted of three RCTs comparing LDCT with usual care [22–24]; one trial comparing LDCT with CXR [8, 25]; and two trials comparing CXR with usual care [26–28]. A further RCT of CXR vs usual care was included in a sensitivity analysis [29]. The estimated relative risk of lung cancer mortality of LDCT compared with usual care was 0.95 (95% CI 0.82 to 1.11) (Additional file 1: Table S5). LDCT was ranked first according to the estimated surface under the cumulative ranking curve values, with a 74.8% probability of being the best intervention in terms of lung cancer mortality reduction. Usual care had a 74.7% probability of being the second best strategy among the three interventions. However, CXR screening had a 99.7% probability of being the worst intervention in terms of lung cancer mortality reduction. Both consistency and inconsistency models were fit for lung cancer mortality data. By applying the design-by-treatment model, we did not find any evidence of inconsistency. The global test for inconsistency gave a *p* value of 0.29, indicating no evidence of inconsistency.

**Fig. 4:**
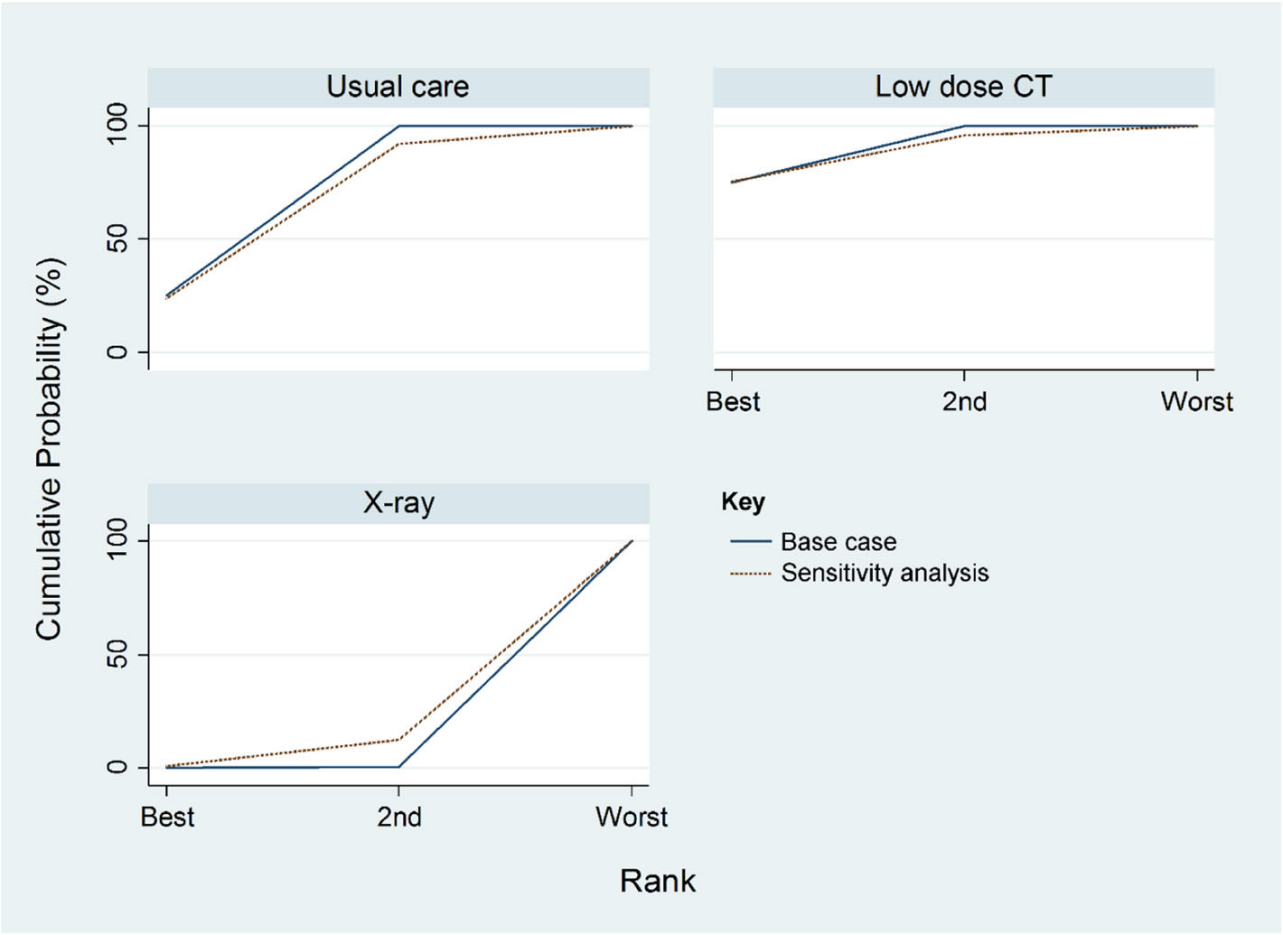
Network meta-analysis rankogram

In the sensitivity analysis, the estimated relative risk of lung cancer mortality comparing LDCT with usual care was 0.93 (95% CI 0.76 to 1.14) (Additional file 1: Table S6). Based on the estimated surface under the cumulative ranking curve values, LDCT screening was ranked first—it had a 75.3% probability of being the best intervention in terms of lung cancer mortality reduction. Usual care had a 68.3% probability of being the second best strategy among the three interventions. Similarly, CXR screening had an 87.7% probability of being the worst intervention in terms of the lung cancer mortality outcome. Again there was no evidence of inconsistency (*p* = 0.18) (Fig. 4).

## Discussion

The main findings of the systematic review and meta-analysis of RCTs comparing LDCT screening programmes with usual care (no screening) or other imaging screening programme (such as chest X-ray (CXR)) are a statistically non-significant decrease in lung cancer mortality (pooled RR 0.94, 95% CI 0.74 to 1.19) and a statistically non-significant increase in all-cause mortality outcome (pooled RR 1.01, 95% CI 0.87 to 1.16). The network meta-analysis agrees with the direct meta-analysis that LDCT is the most effective screening option between LDCT, CXR and usual care, but also raises the possibility that CXR screening may be less effective than usual care. This in turn has implications for the interpretation of the largest RCT which compares LDCT with CXR rather than usual care, the most relevant comparator in trying to decide whether LDCT screening should be introduced.

There is thus considerable uncertainty about the effect of LDCT screening on mortality, particularly the size of the effect. Contributors to this uncertainty beyond the relevance of the main included RCT, are the width of the 95% CI (which include no effect) and the statistical heterogeneity between the included study results. Further, although a RR of 0.94 looks like a useful effect, the rarity of the outcomes in the trials needs to be taken into account (4.7 lung cancer deaths per 100 persons over an 8-year period was found in the Detection And screening of early lung cancer with Novel imaging Technology and molecular Essays (DANTE) RCT [22], which identified the highest lung cancer risk of death in the RCTs contributing data on mortality). This translates to a number needed to screen to avoid one lung cancer death of 357 (95% CI 82 to − 113 [negative value indicates screening increases lung cancer deaths; the confidence interval includes infinity, equivalent to a risk reduction of 0, so the point estimate is encompassed by the interval although apparently lying outside it]) emphasising the considerable number of participants who need to be screened multiply over a period of at least five years to achieve one less lung cancer death even in high-risk populations. The uncertainty is confirmed if the evidence is GRADED, with downgrading for imprecision, inconsistency and indirectness.

The research we report was undertaken by an experienced health technology assessment group, working to a pre-specified protocol, informed by comments from a steering group with wide expertise. The technology assessment as a whole was informed by a patient and public involvement exercise. No members of the research team had any connection with the research teams doing any of the included RCTs. Although likely to increase in the foreseeable future, our main limitation was the small number of included studies. This limited our ability to investigate the heterogeneity between study results and to explore other important phenomena like publication bias. We also did not have opportunity to systematically contact each of the original research teams which may have helped fill some of the gaps in details about the RCTs, particularly randomisation methods. We were conscious that selected enquiry of particular studies might in itself introduce bias.

Our findings are not consistent with recent guidelines by the US Preventive Services Task Force [9] nor with recent expert comment in the UK [37] both of which are strongly supportive of the need to implement LDCT screening for lung cancer. They emphasise the results of the NLST trial pointing to its size and apparent conclusive results. However, we are not alone in pointing to the need to consider the whole evidence base [7, 38]. We have not ignored trials which in normal circumstances would be considered sufficiently important in terms of size and quality to be taken into account in deciding policy. Method of analysis does not appear to be the issue as our pooled estimate of effect on lung cancer mortality just including DANTE, Danish Lung Cancer Screening Trial (DLST) and NLST (excluding MILD) (RR 0.85, 95% CI 0.74 to 0.98) is very similar to the pooled estimate relied on by the US Preventative Task Force, RR 0.81 (95% CI 0.72 to 0.91) [9]. A particular contribution of this study has been to consider the implications of NLST using CXR screening as the comparator. The NLST authors argued qualitatively that CXR screening has no effect using the PLCO NLST high-risk sub-group results and so that NLST gives a valid estimate of LDCT screening versus no screening. We used a formal network meta-analysis, which suggests that CXR screening may possibly be ineffective, so adding a further note of caution about whether NLST may be overestimating the effect of LDCT screening for lung cancer.

## Conclusions

On balance, the evidence does not yet clearly support the case for population LDCT screening and any final decision should await the results of trials in progress. Our recommendation is that the evidence should be re-reviewed when the trials in progress are reported. This should include additional detailed investigation about study quality of all RCTs as we have provisionally identified this as a potential explanatory factor of the heterogeneity between the four RCTs published so far. Up-to-date reviews of other outcomes and cost-effectiveness modelling will also be important aspects of further research. These have already been conducted as part of this project and are reported in a recent publication [13].

## Supplementary information

**Additional file 1.** Web resources. Web **Table S1**-**Table S6**, **Figure S1**-**S2**.

## Data Availability

Data sharing not applicable to this article as no datasets were generated or analysed during the current study.

## Abbreviations

CI: Confidence interval
CT: Computed tomography
CXR: Chest X-ray
DANTE: Detection And screening of early lung cancer with Novel imaging Technology and molecular Essays
DLSCT: Danish Lung Cancer Screening Trial
ITALUNG: Not an abbreviation
LDCT: Low-dose computed tomography
LUSI: German LUng cancer Screening Intervention trial
MILD: Multicentric Italian Lung Detection project
NELSON: NEderlands Leuvens Longkanker Screenings ONderzoek
NLST: National Lung Screening Trial
PLCO: Prostate, Lung, Colorectal and Ovarian cancer screening trial
PRISMA: Preferred Reporting Items for Systematic reviews and Meta-Analyses
RCT: Randomised controlled trial
RR: Relative risk
UKLS: UK Lung cancer Screening trial

## References

[1] World Cancer Research Fund International. Lung cancer statistics. 2017. URL: http://www.wcrf.org/int/cancer-facts-figures/data-specific-cancers/lung-cancer-statistics. Accessed 8 May 2017.

[2] Eurostat. Cancer statistics - specific cancers. 2017. URL: http://ec.europa.eu/eurostat/statistics-explained/index.php/Cancer_statistics_-_specific_cancers#Lung_cancer. Accessed 8 May 2017.

[3] Cancer Research UK. Lung cancer survival statistics. 2017. URL: http://www.cancerresearchuk.org/health-professional/cancer-statistics/statistics-by-cancer-type/lung-cancer/survival. Accessed 8 May 2017.

[4] Chyou PH, Nomura AM, Stemmermann GN (1992). A prospective study of the attributable risk of cancer due to cigarette smoking. Am J Public Health.

[5] Cancer Research UK. Survival. 2017. URL: http://about-cancer.cancerresearchuk.org/about-cancer/lung-cancer/survival. Accessed 8 May 2017.

[6] Royal College of Physicians (2017). National Lung Cancer Audit annual report 2016 (for the audit period 2015).

[7] Manser R, Lethaby A, Irving LB, Stone C, Byrnes G, Abramson MJ (2013). Screening for lung cancer. Cochrane Database Syst Rev.

[8] Aberle DR, Adams AM, Berg CD, Black WC, Clapp JD, National Lung Screening Trial Research Team (2011). Reduced lung-cancer mortality with low-dose computed tomographic screening. N Engl J Med.

[9] Moyer VA, MD, MPH, on behalf of the U.S. Preventive Services Task Force (2014). Screening for Lung Cancer: U.S. Preventive Services Task Force Recommendation Statement. Ann Intern Med.

[10] Black C, Bagust A, Boland A, Walker S, McLeod C, De Verteuil R, et al. The clinical effectiveness and cost-effectiveness of computed tomography screening for lung cancer: systematic reviews. Health Technol Assess. 2006;10(3). 10.3310/hta10030. 10.3310/hta10030 16409881

[11] Black C, de Verteuil R, Walker S, Ayres J (2007). Population screening for lung cancer using computed tomography, is there evidence of clinical effectiveness? A systematic review of the literature. Thorax.

[12] Technology Assessment Report commissioned by the NETSCC HTA HTA 14/151/07. Final PROTOCOL June 2016. URL: https://njl-admin.nihr.ac.uk/document/download/2011033. Accessed 15 Jan 2018.

[13] Snowsill T, Yang H, Griffin E, Long L, Varley-Campbell J, Coelho H (2018). Low-dose computed tomography for lung cancer screening in high-risk populations: a systematic review and economic evaluation. Health Technol Assess.

[14] Higgins JPT, Altman DG, Sterne JAC (editors). Chapter 8: Assessing risk of bias in included studies. In: Higgins JPT, Green S (editors). Cochrane Handbook for Systematic Reviews of Interventions Version 5.1.0. The Cochrane Collaboration; 2011.

[15] DerSimonian R, Laird N (1986). Meta-analysis in clinical trials. Control Clin Trials.

[16] Higgins JPT, Altman DG, Sterne JAC (editors). Chapter 9: Analysing data and undertaking meta-analyses. In: Higgins JPT, Green S (editors). Cochrane Handbook for Systematic Reviews of Interventions Version 5.1.0. The Cochrane Collaboration; 2011.

[17] White IR (2011). Multivariate random-effects meta-regression: updates to mvmeta. Stata J.

[18] Salanti G, Ades AE, Ioannidis JPA (2011). Graphical methods and numerical summaries for presenting results from multiple-treatment meta-analysis: an overview and tutorial. J Clin Epidemiol.

[19] Higgins JPT, Jackson D, Barrett JK, Lu G, Ades AE, White I (2012). R. Consistency and inconsistency in network meta-analysis: concepts and models for multi-arm studies. Res Synth Methods.

[20] Harris RJBM, Deeks JJ, Harbord RM, Altman DG, Sterne JAC (2009). Meta-analysis in Stata: metan, metacum, and metap.

[21] White IR, Barrett JK, Jackson D, Higgins JP (2012). Consistency and inconsistency in network meta-analysis: model estimation using multivariate meta-regression. Res Synth Methods.

[22] Infante M, Cavuto S, Lutman FR, Brambilla G, Chiesa G, Ceresoli G (2009). A randomized study of lung cancer screening with spiral computed tomography: three-year results from the DANTE trial. Am J Respir Crit Care Med.

[23] Pedersen JH, Ashraf H, Dirksen A, Bach K, Hansen H, Toennesen P (2009). The Danish randomized lung cancer CT screening trial--overall design and results of the prevalence round. J Thorac Oncol.

[24] Pastorino U, Rossi M, Rosato V, Marchianò A, Sverzellati N, Morosi C (2012). Annual or biennial CT screening versus observation in heavy smokers: 5-year results of the MILD trial. Eur J Cancer Prev.

[25] Church TR, Black WC, Aberle DR, Berg CD, Clingan KL, National Lung Screening Trial Research Team (2013). Results of initial low-dose computed tomographic screening for lung cancer. N Engl J Med.

[26] Kubik A, Haerting J (1990). Survival and mortality in a randomized study of lung cancer detection. Neoplasma.

[27] Kubık AK, Parkin DM, Zatloukal P (2000). Czech Study on Lung Cancer Screening. Post-trial follow-up of lung cancer deaths up to year 15 since enrollment. Cancer.

[28] Marcus PM, Bergstralh EJ, Fagerstrom RM, Williams DE, Fontana R, Taylor WF (2000). Lung cancer mortality in the Mayo Lung Project: Impact of extended follow-up. J Natl Cancer Inst.

[29] Oken MM, Hocking WG, Kvale PA, Andriole GL, Buys SS, Church TR (2011). Screening by chest radiograph and lung cancer mortality: the Prostate, Lung, Colorectal, and Ovarian (PLCO) randomized trial. JAMA.

[30] Lopes Pegna A, Picozzi G, Falaschi F, Carrozzi L, Falchini M, Carozzi FM (2013). Four-year results of low-dose CT screening and nodule management in the ITALUNG trial. J Thorac Oncol.

[31] Becker N, Motsch E, Gross ML, Eigentopf A, Heussel CP, Dienemann H (2012). Randomized study on early detection of lung cancer with MSCT in Germany: study design and results of the first screening round. J Cancer Res Clin Oncol.

[32] Horeweg N, Scholten ET, de Jong PA, van der Aalst CM, Weenink C, Lammers JWJ (2014). Detection of lung cancer through low-dose CT screening (NELSON): A prespecified analysis of screening test performance and interval cancers. Lancet Oncol.

[33] Horeweg N, van der Aalst CM, Vliegenthart R, Zhao Y, Xie X, Scholten ET (2013). Volumetric computed tomography screening for lung cancer: three rounds of the NELSON trial. Eur Respir J.

[34] Field JK, Duffy SW, Baldwin DR, Brain KE, Devaraj A, Eisen T (2016). The UK Lung Cancer Screening Trial: a pilot randomised controlled trial of low-dose computed tomography screening for the early detection of lung cancer. Health Technol Assess.

[35] Begg C, Cho M, Eastwood S, Horton R, Moher D, Olkin I (1996). Improving the quality of reporting of randomized controlled trials. The CONSORT Statement. JAMA.

[36] Marcus PM, Prorok PC (1999). Reanalysis of the Mayo Lung Project data: the impact of confounding and effect modification. J Med Screen.

[37] Baldwin DR, ten Haaf K, Rawlinson J, Callister MEJ (2017). Low dose CT screening for lung cancer. Pilots in car parks take the UK one step closer to national screening. BMJ.

[38] Ali MU, Miller J, Peirson L, Fitzpatrick-Lewis D, Kenny M, Sherifali D (2016). Screening for lung cancer: a systematic review and meta-analysis. Preventive Med.

