# Supplementary material for: Do we know enough about the effect of low-dose computed tomography screening for lung cancer on survival to act? A systematic review, meta-analysis and network meta-analysis of randomised controlled trials
Source: Diagn Progn Res. 2019 Nov 28;3:23. doi: 10.1186/s41512-019-0067-4 (PMC6933743; doi:10.1186/s41512-019-0067-4)

**Web Sources**

Web Table 1 Example search strategy for MEDLINE

Lung cancer screening searches—clinical effectiveness 1, 2004-Jan 2012, no comparator, low dose CT only, RCT filter

Database: MEDLINE

Host: Ovid

Data Parameters: 1946 to December week 1 2016

Date Searched: 9/1/2017

Searcher: SR

Hits: 183

1. exp Lung Neoplasms/

2. ((lung$ or bronch$ or pulmon$) adj3 (cancer$ or neopla$ or tumor$ or tumour$ or carcinoma$ or adenocarcinoma$ or small cell or squamous)).ti,ab,ot,kw.

3. (NSLC or NSCLC or SLC or SCLC).ti,ab,ot,kw.

4. 1 or 2 or 3

5. exp Tomography, X-Ray Computed/

6. ((CT or CAT) adj3 (scan$ or screen$)).ti,ab,ot,kw.

7. ((computer$ adj3 tomogra$) and (scan$ or screen$)).ti,ab,ot,kw.

8. (tomogra$ or helix or helical or spiral$ or spiro$).ti,ab,ot,kw.

9. 5 or 6 or 7 or 8

10. ((low$ adj3 dos$) or LDCT).ti,ab,kw,ot.

11. ((ultralow$ or ultra-low$) adj3 dos$).ti,ab,kw,ot.

12. (low-dos$ or ultralow-dos$).ti,ab,kw,ot.

13. 10 or 11 or 12

14. randomized controlled trial.pt.

15. controlled clinical trial.pt.

16. randomized.ab.

17. placebo.ab.

18. drug therapy.fs.

19. randomly.ab.

20. trial.ab.

21. groups.ab.

22. 11 or 12 or 13 or 14 or 15 or 16 or 17 or 18

23. exp animals/ not humans.sh.

24. 19 not 20

25. 4 and 9 and 13 and 24

26. (200407* or 200408* or 200409* or 200410* or 200411* or 200412* or 2005* or 2006* or 2007* or 2008* or 2009* or 2010* or 2011* or 201201*).ed.

27. 25 and 26

28. Limit 27 to English language and yr=”2004-“

Lung cancer screening searches – clinical effectiveness 2, 2012- current, all CT scan doses, X-ray comparator, RCT filter

Database: MEDLINE

Host: Ovid

Data Parameters: 1946 to December week 1 2016

Date Searched: 10/1/2017

Searcher: SR

Hits: 2074

1. exp Lung Neoplasms/

2. ((lung$ or bronch$ or pulmon$) adj3 (cancer$ or neopla$ or tumor$ or tumour$ or carcinoma$ or adenocarcinoma$ or small cell or squamous)).ti,ab,ot,kw.

3. (NSLC or NSCLC or SLC or SCLC).ti,ab,ot,kw.

4. 1 or 2 or 3

5. exp Tomography, X-Ray Computed/

6. exp Radiography, Thoracic/

7. (x ray or xray or x-ray or CXR or radiograph$).ti,ab,ot,kw.

8. ((CT or CAT) adj3 (scan$ or screen$)).ti,ab,ot,kw.

9. ((computer$ adj3 tomogra$) and (scan$ or screen$)).ti,ab,ot,kw.

10. (tomogra$ or helix or helical or spiral$ or spiro$).ti,ab,ot,kw.

11. 5 or 6 or 7 or 8 or 9 or 10

12. 4 and 11

13. (2012* or 2013* or 2014* or 2015* or 2016* or 2017*).ed.

14. randomized controlled trial.pt.

15. controlled clinical trial.pt.

16. randomized.ab.

17. placebo.ab.

18. drug therapy.fs.

19. randomly.ab.

20. trial.ab.

21. groups.ab.

22. 14 or 15 or 16 or 17 or 18 or 19 or 20 or 21

23. exp animals/ not humans.sh.

24. 22 not 23

25. 12 and 13 and 24

26. limit 25 to english language and yr=”2012-Current”

Web Table 2 Outcomes in included studies in qualitative systematic review of RCTs of low dose CT screening for lung cancer

Web table 2. Outcomes in included studies in qualitative systematic review of RCTs of low dose CT screening for lung cancer

| Study (recruitment period) | N^A^ | Lung cancer mortality | All-cause mortality | Cancer incidence | Stage distribution | Complete resection | Health related quality of life | Smoking cessation | Additional information |
| --- | --- | --- | --- | --- | --- | --- | --- | --- | --- |
| DANTE^a^  (03/2001 to 02/2006) | 2450 | ≥5y | ≥5y | ≥5y | ≥5y | ≥5y | NR | ≥5y | NCT00420862   - No protocol found - Update (5/2017) combined with MILD |
| Depiscan^b^  (10/2002 to 12/2004) | 830 | NR | NR | NR | NR | NR | NR | NR | - Pilot - No protocol found |
| DLCST^c^  (10/2004 to 03/2006) | 4104 | ≥5y  1^0^ 10y | ≥5y | ≥5y  2^0^ 5y | ≥5y  2^0^ 5y | NR | Consequences of screening lung cancer (COS-LC) 1-5y | Annual smoking status 1-5y | NCT00496977   - Details in clinicaltrials.gov |
| Garg et al 2002^d^  (01/2001 to 10/2001) | 190 | NR | NR | NR | NR | NR | NR | NR | - Feasibility study - No protocol found |
| ITALUNG^e^  (03/2004 to 09/2010 (end of last intervention scan at Y4)) | 3206 | NR  1^0^ 8y | NR  2^0^ 8y | NR  2^0^ 8y^B^ | NR | NR | NR | NR | NCT02777996   - Details in clinicaltrials.gov - Italian protocol at <http://www.ispo.it> - Results >5y recently published but, after inclusion date for this review |
| LSS-PLCO^f^  (09/2000 to 11/2000 or 01/2001)^C^ | 3318 | NR | NR | NR | NR | NR | NR | NR | NCT00006382   - No protocol found - Feasibility study for NSLT |
| LungSEARCH^g^  (08/2007 to 03/2011) | 1568 | NR  2^0^ 15y | NR | NR | NR  1^0^ 5y | NR | NR | NR | NCT00512746   - Details in clinicaltrials.gov |
| LUSI^h^  (09/2007 to 04/2011) | 4052 | NR | NR | NR | NR | NR | NR | NR | - No protocol found - Conference abstract 2016 indicates results imminent |
| MILD^i^  (09/2005 to 09/2011) | 4099 | ≥5y  1^0^ 10y | ≥5y | ≥5y | NR | NR | NR | NR  2^0^ 10y | NCT02837809   - Details in clinicaltrials.gov - Update (5/2017) combined with DANTE |
| NELSON^j,k^  (01/2004 to12/2006) | 15822 | NR  1^0^ | NR | NR | NR | NR | <5y^D^  2^0^ | <5y^D^ | ISRCTN63545820   - Details in ISRCT entry |
| NLST^l,m,n^  (08/2002 to 04/2004) | 53454 | ≥5y^E^  1^0^ | ≥5y^E^  2^0^ | ≥5y^E^  2^0^ | ≥5y | ≥5y | <1y | NR | NCT00047385   - Details in clinicaltrials.gov |
| UKLS^o^  (08/2011 to 08/2012) | 4061 | NR | NR | NR | NR | NR | <5y | NR | ISRCTN78513845   - Stated intention to combine mortality and incidence data with NELSON |
| **Abbreviations:** 1^0^, primary outcome; 2^0^, secondary outcome; N, number; NR, not reported; y, years  **Footnotes:** A, randomised participants; B, including overdiagnosis; C, recruitment period varies according to source; D, subsample of participants; E, pre-specified - all events to 31/12/2009  **References:** | | | | | | | | | |
| a Infante M, Cavuto S, Lutman FR, Brambilla G, Chiesa G, Ceresoli G, et al. A randomized study of lung cancer screening with spiral computed tomography: three-year results from the DANTE trial. Am J Respir Crit Care Med 2009;180(5):445-53. | | | | | | | | | |
| b Blanchon T, Brechot JM, Grenier PA, Ferretti GR, Lemarie E, Milleron B, et al. Baseline results of the Depiscan study: a French randomized pilot trial of lung cancer screening comparing low dose CT scan (LDCT) and chest X-ray (CXR). Lung Cancer 2007;58(1):50-8. | | | | | | | | | |
| c Pedersen JH, Ashraf H, Dirksen A, Bach K, Hansen H, Toennesen P, et al. The Danish randomized lung cancer CT screening trial--overall design and results of the prevalence round. J Thorac Oncol 2009;4(5):608-14. | | | | | | | | | |
| d Garg K, Keith RL, Byers T, Kelly K, Kerzner AL, Lynch DA, et al. Randomized controlled trial with low-dose spiral CT for lung cancer screening: Feasibility study and preliminary results. Radiology 2002;225(2):506-10. | | | | | | | | | |
| e Lopes Pegna A, Picozzi G, Falaschi F, Carrozzi L, Falchini M, Carozzi FM, et al. Four-year results of low-dose CT screening and nodule management in the ITALUNG trial. J Thorac Oncol 2013;8(7):866-75. | | | | | | | | | |
| f Gohagan JK, Marcus PM, Fagerstrom RM, Pinsky PF, Kramer BS, Prorok PC, et al. Final results of the Lung Screening Study, a randomized feasibility study of spiral CT versus chest X-ray screening for lung cancer. Lung Cancer 2005;47(1):9-15. | | | | | | | | | |
| g Spiro SG, Hackshaw A, Lung Search Collaborative Group. Research in progress--LungSEARCH: a randomised controlled trial of surveillance for the early detection of lung cancer in a high-risk group. Thorax 2016;71(1):91-3. | | | | | | | | | |
| h Becker N, Motsch E, Gross ML, Eigentopf A, Heussel CP, Dienemann H, et al. Randomized study on early detection of lung cancer with MSCT in Germany: study design and results of the first screening round. J Cancer Res Clin Oncol 2012;138(9):1475-86. | | | | | | | | | |
| I Pastorino U, Rossi M, Rosato V, Marchianò A, Sverzellati N, Morosi C, et al. Annual or biennial CT screening versus observation in heavy smokers: 5-year results of the MILD trial. Eur J Cancer Prev 2012;21(3):308-15. | | | | | | | | | |
| j Horeweg N, Scholten ET, de Jong PA, van der Aalst CM, Weenink C, Lammers JWJ, et al. Detection of lung cancer through low-dose CT screening (NELSON): A prespecified analysis of screening test performance and interval cancers. Lancet Oncol 2014;15(12):1341-50. | | | | | | | | | |
| k Volumetric computed tomography screening for lung cancer: three rounds of the NELSON trial. Eur Respir J 2013;42(6):1659-67. | | | | | | | | | |
| l Aberle DR, Adams AM, Berg CD, Black WC, Clapp JD, Fagerstrom RM, et al. Reduced lung-cancer mortality with low-dose computed tomographic screening. N Engl J Med 2011;365(5):395-409. | | | | | | | | | |
| m National Lung Screening Trial Research Team, Church TR, Black WC, Aberle DR, Berg CD, Clingan KL, et al. Results of initial low-dose computed tomographic screening for lung cancer. N Engl J Med 2013;368(21):1980-91. | | | | | | | | | |
| n Gareen IF, Duan F, Greco EM, Snyder BS, Boiselle PM, Park ER, et al. Impact of lung cancer screening results on participant health-related quality of life and state anxiety in the National Lung Screening Trial. Cancer 2014;120(21):3401-9. | | | | | | | | | |
| o Field JK, Duffy SW, Baldwin DR, Brain KE, Devaraj A, Eisen T, et al. The UK Lung Cancer Screening Trial: a pilot randomised controlled trial of low-dose computed tomography screening for the early detection of lung cancer. Health Technol Assess 2016;20(40):1-146. | | | | | | | | | |

Web table 3. Balance/imbalance in baseline characteristics between the four low dose CT RCTs included in quantitative meta-analysis

| **Study Characteristics** | **DANTE**^a^ | | **DLSCT**^b^ | | **MILD**^c^ | | | **NLST**^d^ | |
| --- | --- | --- | --- | --- | --- | --- | --- | --- | --- |
| N | 2450 | | 4104 | | 4099 | | | 53456 | |
| Trial arm | LDCT | Control | LDCT | Control | LDCT (biennial) | LDCT (annual) | Control | LDCT | Control |
| n (% of N) | 1264 (51.6) | 1186 (48.4) | 2052 (50) | 2052 (50) | 1186 (28.9) | 1190 (29.0) | 1723 (42.0) | 26723 (50) | 26733 (50) |
| Sex (% of n male) | NR | NR | 55.9^A^ | 54.6^A^ | 68.5 | 68.4 | 63.3 | 59.0 | 59.0 |
| Age (mean y) | 64.6 | 64.6 | 57.9^A^ | 57.9^A^ | 58.2^A^ | 58.3^A^ | 57.6^A^ | 61.6^A^ | 61.6^A^ |
| Occupational exposure (% of n) | 31.3 | 34.1 | NR | NR | NR | NR | NR | 27.9 | 28.3 |
| **Smoking** | | | | | | | | | |
| Current smokers (%) | 56.5 | 57.4 | 75.3^A^ | 76.9^A^ | 68.3 | 68.9 | 89.7 | 48.2 | 48.3 |
| Pack-years (mean) | 47.3 | 47.2 | NR | NR | 39^B^ | 39^B^ | 38^B^ | 56.0 | 55.9 |
| Smoking duration (mean y) | NR | NR | 38.5^A^ | 38.6^A^ | 38.4^A^ | 38.3^A^ | 38.5^A^ | 43.1 | 43.1 |
| Cigs/day (mean) | NR | NR | 19.2^A^ | 18.6^A^ | 26.3^A^ | 26.8^A^ | 25.2^A^ | 28.5 | 28.4 |
| Duration smoking cessation in former smokers (mean y) | NR | NR | 4.2^A^ | 4.4^A^ | NR | NR | NR | 7.7^A^ | 7.7^A^ |
| **Comorbidities** | | | | | | | | | |
| Respiratory | 35.3 | 31.2 | NR | NR | NR | NR | NR | NR | NR |
| Chronic bronchitis, emphysema or COPD | NR | NR | NR | NR | NR | NR | NR | 17.5 | 17.4 |
| Hypertension | 36.1 | 37.7 | NR | NR | NR | NR | NR | 35.1 | 35.7 |
| Cardiac | 12.6 | 13.9 | NR | NR | NR | NR | NR | NR | NR |
| Heart disease or heart attack | NR | NR | NR | NR | NR | NR | NR | 12.9 | 12.5 |
| Stroke | NR | NR | NR | NR | NR | NR | NR | 2.8 | 2.8 |
| PVD | 10.3 | 9.0 | NR | NR | NR | NR | NR | NR | NR |
| Diabetes | 8.3 | 8.4 | NR | NR | NR | NR | NR | 9.7 | 9.7 |
| Malignancies | NR | NR | NR | NR | NR | NR | NR | 4.0 | 4.5 |
| **Lung function** | | | | | | | | | |
| FEV1 (L) | NR | NR | 2.9 | 2.9 | NR | NR | NR | NR | NR |
| FEV1 <90% predicted | NR | NR | NR | NR | 27.7 | 28.2 | 19.2 | NR | NR |
| Other data available |  | | Social status | | Paper indicates that only “selected baseline characteristics” were reported | | | Race; education; marital status; BMI categories | |

**Abbreviations:** NR, not reported; PVD, peripheral vascular disease; FEV, forced expiratory volume.

**Footnotes:** A, calculated by PenTAG; B, median rather than mean Italics indicate where entries calculated from data in paper.

**References:**

a Infante M, Cavuto S, Lutman FR, Passera E, Chiarenza M, Chiesa G, et al. Long-Term Follow-up Results of the DANTE Trial, a Randomized Study of Lung Cancer Screening with Spiral Computed Tomography. Am J Respir Crit Care Med 2015;191(10):1166-75.

b Pedersen JH, Ashraf H, Dirksen A, Bach K, Hansen H, Toennesen P, et al. The Danish randomized lung cancer CT screening trial--overall design and results of the prevalence round. J Thorac Oncol 2009;4(5):608-14.

c Pastorino U, Rossi M, Rosato V, Marchianò A, Sverzellati N, Morosi C, et al. Annual or biennial CT screening versus observation in heavy smokers: 5-year results of the MILD trial. Eur J Cancer Prev 2012;21(3):308-15.

d National Lung Screening Trial Research Team, Aberle DR, Adams AM, Berg CD, Black WC, Clapp JD et al. Reduced lung-cancer mortality with low-dose computed tomographic screening. N Engl J Med 2011;365(5):395-409.

Web table 4. Event data for included studies

| **Study** | **Comparator** | **Lung cancer mortality** | | | | **All-cause mortality** | | | |
| --- | --- | --- | --- | --- | --- | --- | --- | --- | --- |
|  |  | Number of events | Total number of participants | Number of events | Total number of participants | Number of events | Total number of participants | Number of events | Total number of participants |
|  |  | Low dose CT group | | Control group | | Low dose CT group | | Control group | |
| DANTE^a^ | Usual care | 59 | 1264 | 55 | 1186 | 180 | 1264 | 176 | 1186 |
| DLCST^b^ | Usual care | 39 | 2052 | 38 | 2052 | 165 | 2052 | 163 | 2052 |
| MILD^c^ (Annual) | Usual care | 12 | 1190 | 7 | 1723 | 31 | 1190 | 20 | 1723 |
| MILD^c^ (Biannual) | Usual care | 6 | 1186 |  |  | 20 | 1186 |  |  |
| NLST^d^ | CXR | 356 | 26722 | 443 | 26732 | 1877 | 26722 | 2000 | 26732 |
|  |  |  |  |  |  |  |  |  |  |
|  |  | CXR group | | Control group | |  | |  | |
| Czech^e^ | Single CXR | 247 | 3172 | 216 | 3174 |  |  |  |  |
| MAYO^f^ | Usual care | 337 | 4607 | 303 | 4585 |  |  |  |  |
| PLCO^g^ (NLST eligible sub-group) | No screening | 316 | 15183 | 334 | 15138 |  |  |  |  |
|  |  |  |  |  |  |  |  |  |  |
| **References:** | | | | | | | | | |
| a Infante M, Cavuto S, Lutman FR, Brambilla G, Chiesa G, Ceresoli G, et al. A randomized study of lung cancer screening with spiral computed tomography: three-year results from the DANTE trial. Am J Respir Crit Care Med 2009;180(5):445-53. | | | | | | | | | |
| b Pedersen JH, Ashraf H, Dirksen A, Bach K, Hansen H, Toennesen P, et al. The Danish randomized lung cancer CT screening trial--overall design and results of the prevalence round. J Thorac Oncol 2009;4(5):608-14. | | | | | | | | | |
| c Pastorino U, Rossi M, Rosato V, Marchianò A, Sverzellati N, Morosi C, et al. Annual or biennial CT screening versus observation in heavy smokers: 5-year results of the MILD trial. Eur J Cancer Prev 2012;21(3):308-15. | | | | | | | | | |
| d National Lung Screening Trial Research Team, Aberle DR, Adams AM, Berg CD, Black WC, Clapp JD et al. Reduced lung-cancer mortality with low-dose computed tomographic screening. N Engl J Med 2011;365(5):395-409. | | | | | | | | | |
| e Kubık AK, Parkin DM, Zatloukal P. Czech Study on Lung Cancer Screening. Post-trial follow-up of lung cancer deaths up to year 15 since enrollment. Cancer 2000;89:2363–8. | | | | | | | | | |
| f Marcus PM, Bergstralh EJ, Fagerstrom RM, Williams DE, Fontana R, Taylor WF, et al. Lung cancer mortality in the Mayo Lung Project: Impact of extended follow-up. J Natl Cancer Inst 2000;92(16):1308-16. | | | | | | | | | |
| g Oken MM, Hocking WG, Kvale PA, Andriole GL, Buys SS, Church TR, et al. Screening by chest radiograph and lung cancer mortality: the Prostate, Lung, Colorectal, and Ovarian (PLCO) randomized trial. JAMA 2011;306(17):1865-73. | | | | | | | | | |

Web table 5. Network meta-analysis results for relative risks of lung cancer mortality for pairwise comparisons - Overall results

|  | **Relative risk (RR)** | **95% Confidence Interval** |
| --- | --- | --- |
| Low-dose CT vs. usual care | 0.95 | 0.82 to 1.11 |
| X-ray vs. usual care | 1.15 | 1.03 to 1.28 |
| Low-dose CT vs. X-ray | 0.83 | 0.73 to 0.94 |

Web table 6. Network meta-analysis results for relative risks of lung cancer mortality for pairwise comparisons - Sensitivity analysis

|  | **Relative risk (RR)** | **95% Confidence Interval** |
| --- | --- | --- |
| Low-dose CT vs. usual care | 0.93 | 0.76 to 1.14 |
| X-ray vs. usual care | 1.09 | 0.97 to 1.22 |
| Low-dose CT vs. X-ray | 0.86 | 0.72 to 1.03 |

Web Figure 1. Lung cancer mortality – sensitivity analysis by excluding the low quality trial (MILD)


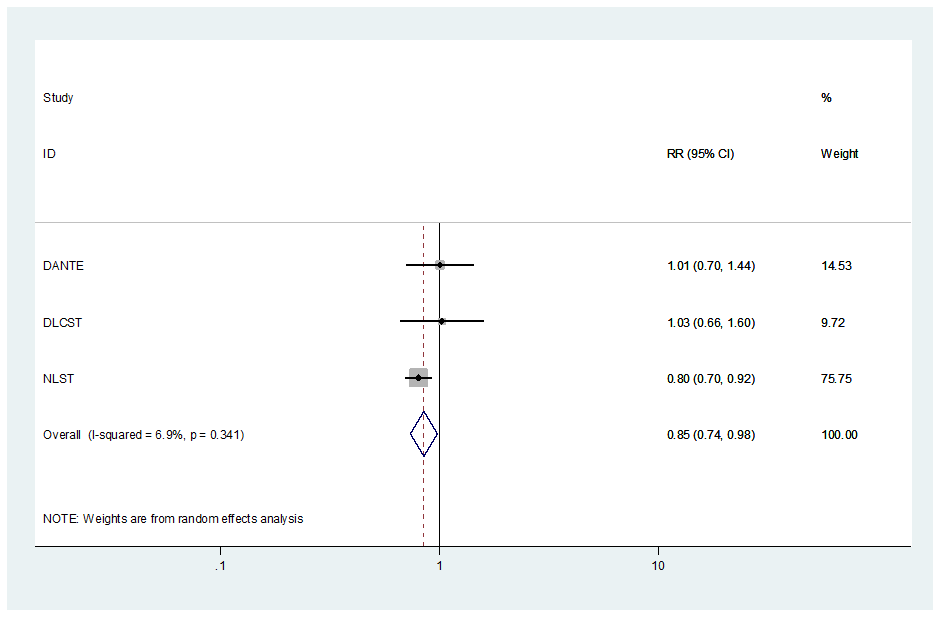


Web Figure 2. All cause mortality – sensitivity analysis by excluding the low quality trial (MILD)


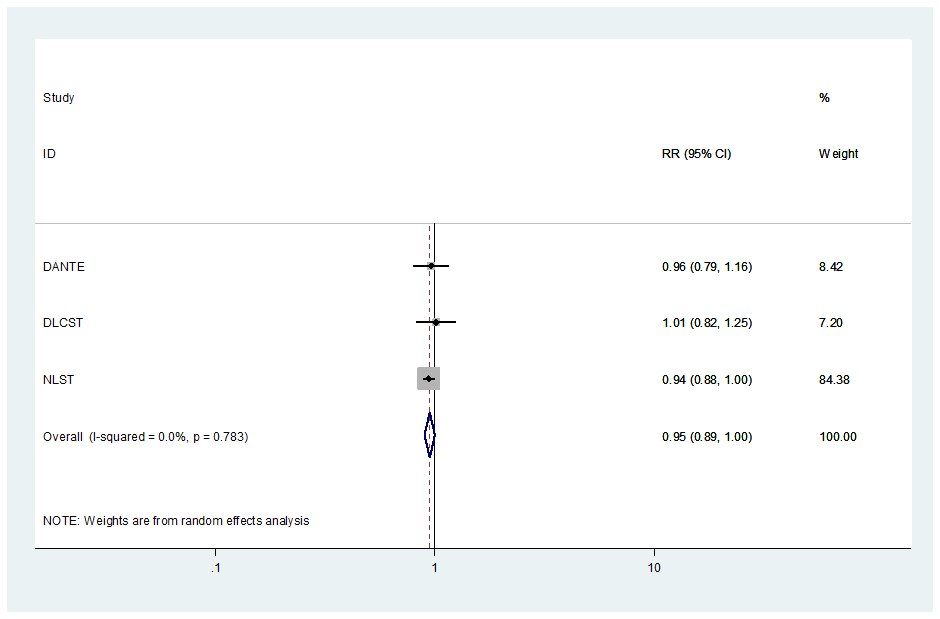


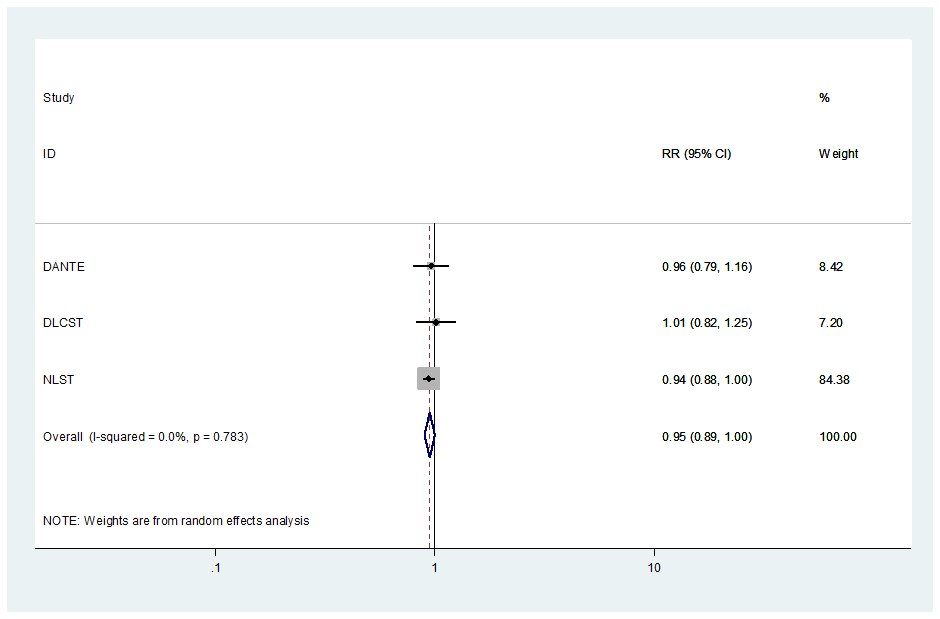

Supplement: Supplementary file 1 — Additional file 1. Web resources. Web Table S1-Table S6, Figure S1-S2. [file 41512_2019_67_MOESM1_ESM.docx]
